# Superior properties of CellTrace Yellow™ as a division tracking dye for human and murine lymphocytes

**DOI:** 10.1111/imcb.1020

**Published:** 2017-12-15

**Authors:** Jessica C Tempany, Jie HS Zhou, Philip D Hodgkin, Vanessa L Bryant

**Affiliations:** ^1^ Division of Immunology The Walter and Eliza Hall Institute of Medical Research Parkville VIC Australia; ^2^ Department of Medical Biology The University of Melbourne Parkville VIC Australia

**Keywords:** Cell biology, cell division, cell imaging, division tracking, flow cytometry, human lymphocytes, murine lymphocytes

## Abstract

The discovery of cell division tracking properties of 5‐(and‐6)‐carboxyfluorescein diacetate succinimidyl ester (CFSE) by Lyons and Parish in 1994 led to a broad range of new methods and numerous important biological discoveries. After labeling, CFSE is attached to free amine groups and intracellular proteins in the cytoplasm and nucleus of a cell, and halves in fluorescence intensity with each round of cell division, enabling enumeration of the number of divisions a cell has undergone. A range of popular division tracking dyes were subsequently developed, including CellTrace Violet (CTV), making available the green fluorescent channel previously occupied by CFSE. More recently, CellTrace Yellow (CTY) and CellTrace Far Red (CTFR), each with unique fluorescence properties, were introduced. In a comparison, we found that the fluorescence values of both dyes were well separated from autofluorescence, and enabled a greater number of divisions to be identified than CTV, before this limit was reached. These new dyes provided clear and well‐separated peaks for both murine and human B lymphocytes, and should find wide application. The range of excitation/emission spectra available for division tracking dyes now also facilitates multiplexing, that is, the labeling of cells with different combinations of dyes to give a unique fluorescence signature, allowing single cell *in vitro* and *in vivo* tracking. The combinatorial possibilities are significantly increased with these additional dyes.

## Introduction

The immune system is a complex and carefully‐regulated environment where lymphocytes coordinate their proliferation, differentiation, survival and migration behavior to orchestrate an appropriate response. The use of fluorescent dye‐labeling to interrogate ongoing lymphocyte responses, both *in vitro* and *in vivo* has led to significant advances in our understanding of immune regulation.^1^, ^2^, ^3^ The most widely used division tracking dye method to date, labeling cells with 5‐(and‐6)‐carboxyfluorescein diacetate succinimidyl ester (CFSE), was introduced in 1994 by Lyons and Parish.^1^ This dye incorporates into both the cytoplasm and nucleus of cells, and is well‐retained within stained cells.^4^ Upon division, CFSE distributes evenly between daughter cells, resulting in a twofold dilution with each consecutive cell division, forming distinct peaks when a proliferating population is viewed as a histogram following flow cytometry analysis.^1^ This method enables determination of the number of divisions each cell has undergone, until the CFSE fluorescence is too dilute to be distinguishable from autofluorescence of the cell. CFSE is not passed to neighboring cells, due to the covalent bonds between succinimidyl esters and proteins. Use of CFSE, however, is somewhat limited by its incompatibility with green fluorescent protein, thus excluding its utility for *in vivo* cell‐tracking in many reporter animal models. Other chemicals have since become available to be used for division tracking of cells in place of, or in combination with, CFSE, such as SNARF^5^, ^6^ and PKH dyes. However, the reduced intensity of labeling^5^, ^6^ and poor definition of division peaks of these dyes have limited their applications.^7^

In recent years, additional fluorochrome‐based tracking dyes have become available. CellTrace Violet (CTV) was developed with an excitation range suited to the violet laser (405 nm), enabling concurrent use of the most frequently‐used fluorescence channels, such as those for green fluorescent protein and phycoerythrin. A further advantage over CFSE was that CTV requires little spectral compensation with many fluorochromes used in flow cytometry, and the initial staining intensity was consistently narrow among homogenous cells,^8^ allowing better segregation of generations upon subsequent analyses.

Many notable discoveries have taken advantage of division tracking dyes. These include the discoveries that T and B lymphocytes share a common regulatory process where changes in class of the response are linked to clonal expansion, and that cell division times are variable and stochastic, but highly concordant in families.^9^, ^10^, ^11^, ^12^, ^13^, ^14^, ^15^, ^16^, ^17^ These dyes have also been used to study molecular symmetry at mitosis, and to investigate asymmetric cell division in lymphocytes.^18^, ^19^, ^20^ CFSE has been used in several studies, alone or in combination with other dyes, to investigate cytotoxic T cell killing or regulatory T cell suppression of target cells,^6^, ^21^, ^22^, ^23^, ^24^ as well as in the investigation of NK cell proliferation regulation.^25^ Most recently, CTV was used to examine the differentiation of CD8 +  T cells into the effector and memory cell pools during influenza infection,^26^ and to demonstrate a division‐independent, timed control of lymphocyte proliferation.^27^ Division tracking dyes are not restricted to lymphocytes, but can also be used to label other cell types such as hematopoietic stem cells.^28^, ^29^

A limiting factor for both CFSE and CTV is that they emit in the 450–550 nm range (Table 1), where autofluorescence, due in large part to pyridinic and flavin coenzymes, aromatic amino acids and lipo‐pigments, increases in larger and activated cells.^8^, ^30^, ^31^ Here, we wish to highlight the advantages of two new cell tracking dyes, CellTrace Yellow (CTY) and CellTrace Far Red (CTFR, Thermo Fisher Scientific, MA, USA.), and to discuss specific advantages of their use for the study of both murine and human lymphocytes.

**Table 1:** Excitation wavelengths, emission filters, and concentrations of division tracking dyes tested

Division tracking dye	Excitation laser (nm)	Emission detection (nm)	Concentration used (μmol L^−1^)
CellTrace Violet (CTV)	405	425–475	2.5–10
CellTrace Yellow (CTY)	561	578–594	5–20
CellTrace Far Red (CTFR)	635	655–685	1–8

## Results

### Comparison of division tracking dyes reveals high intensity of staining and reduced toxicity for CTY‐labeled lymphocytes

**Figure 1:**
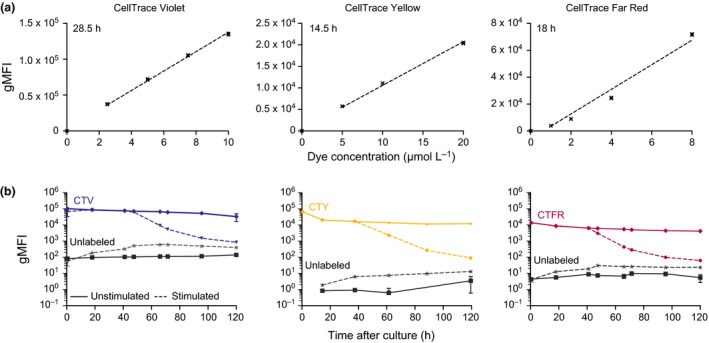
Comparison of labeling intensity for three division tracking dyes. Purified naïve murine splenic B cells were labeled as indicated. **(a)** The gMFI of cells labeled with CTV, CTY, or CTFR are shown relative to the concentrations tested for labeling, when harvested prior to first division (at 28 h, 14.5 h and 18 h, respectively). Lines of best fit are overlaid. **(b)** Comparison between gMFI for cells harvested daily, that were either unlabeled or labeled with CTV (5 μmol L^−1^), CTY (20 μmol L^−1^), or CTFR (2 μmol L^−1^). Cells were cultured in the presence or absence of anti‐CD40 antibody and IL‐4 (stimulated or unstimulated, respectively). Average gMFI values ± SEM of triplicate wells for each cell tracking dye are shown. Results are representative of at least 2 independent titrations per dye. CTY, CellTrace Yellow; CTV, CellTrace Violet; CTFR, CellTrace Far Red; gMFI, geometric mean fluorescence intensities.

Murine naïve B cells were labeled with a range of concentrations of CTV, CTY or CTFR, to test for dye uptake and toxicity. Labeled cells were cultured in the presence of 10 μg mL^−1^ anti‐CD40 agonistic antibody (clone 1C10) and 500 U mL^‐1^ recombinant murine IL‐4, and fluorescence intensity followed over time. Label efficiency was measured after at least 30 min of culture to allow the dye to stabilize. For all three dyes, we observed a linear relationship between dye concentration and fluorescence level measured (Figure 1a). This suggests that a higher initial concentration of division tracking dye will allow a greater number of division peaks to be detected before merging with the background level of autofluoresence (Supplementary figure 1a). However, in practice, application of this principle is limited by the toxicity of the dye and/or the final level of dimethyl sulfoxide (DMSO) solvent used with each dye. Compared to unlabeled cells, CTV labeling of cells at 5 μmol L^−1^ (manufacturer's recommended starting concentration) is not toxic (as measured by total cell number vs. dose, Supplementary figure 1b), whereas when used above 7.5 μmol L^−1^, CTV has a toxic effect on murine naïve B cells. CTY at 5, 10 (manufacturer's recommended starting concentration), or 20 μmol L^−1^ has little observed toxicity on murine naïve B cells (Supplementary figure 1b), when compared to unlabeled cells. CTFR labeling of cells resulted in a decrease in cell number for concentrations above 2 μmol L^−1^ (manufacturers’ recommended concentration is 1 μmol L^−1^; Supplementary figure 1b), indicating cell sensitivity at these concentrations and above.

### Lower background autofluorescence of cells in CTY channel, compared to CTV and CTFR

CTV, CTY and CTFR labeling was well‐retained over time for each dye (Figure 1b). A notable feature, and point of difference, between CTY and CTFR compared to CTV, is that both resting and activated B cells have extremely low background fluorescence levels when cytometer voltages were optimized for labeled cells in the relevant detection channels (Figure 1b). This was highlighted by the number of decades that separated autofluorescence in unlabeled cells and undivided, labeled cells in each channel: 3 decades for CTV, compared to 4–5 for CTY, and 3–4 for CTFR. All detector channels recorded an increase in autofluorescence for unlabeled, stimulated cells over time, likely due to an increase in cytoplasm and cell size upon stimulation. However, this change had the largest impact in the CTV channel, where the effective range of the dye was further reduced to just over 2 decades. The conclusion that CTY and CTFR maintained a greater dynamic range in the late stages of the experiments was confirmed with the Staining Index calculations of Maecker *et al.,* 32 which accounts not only for the distance between labeled and unlabeled fluorescence values, but also the variance of unlabeled cells. Both CTY and CTFR gave greater sensitivity than CTV by 61.5 h after stimulation (Supplementary table 1). In contrast, background fluorescence of unlabeled, unstimulated cells remained consistently low over the course of the culture in all three channels.

The significantly lower autofluorescence of resting and activated cells in CTY and CTFR channels offers a broader range of fluorescence values to detect dividing cells, thus allowing for the resolution of a greater number of generations. The dynamic range of CTY could potentially be even further expanded as CTY may be used at higher concentrations (up to 40 μmol L^−1^) without observing any toxicity, whereas CTV is toxic above 7.5 μmol L^−1^ and CTFR above 4 μmol L^−1^ (Supplementary figure 1b).

Quah *et al*. recently reported that toxicity when labeling cells with division tracking dyes can be reduced by adding serum to the labeling media, enabling users to label cells with up to 120 μmol L^−1^ of cell tracking dye.^8^ While we also observed reduced cell loss during the labeling protocol using this method, in our hands, this resulted in significantly reduced labeling efficiency per volume of dye (data not shown).

### Resolution of peaks is clearly defined for cells labeled with CTV and CTY, but not CTFR

**Figure 2:**
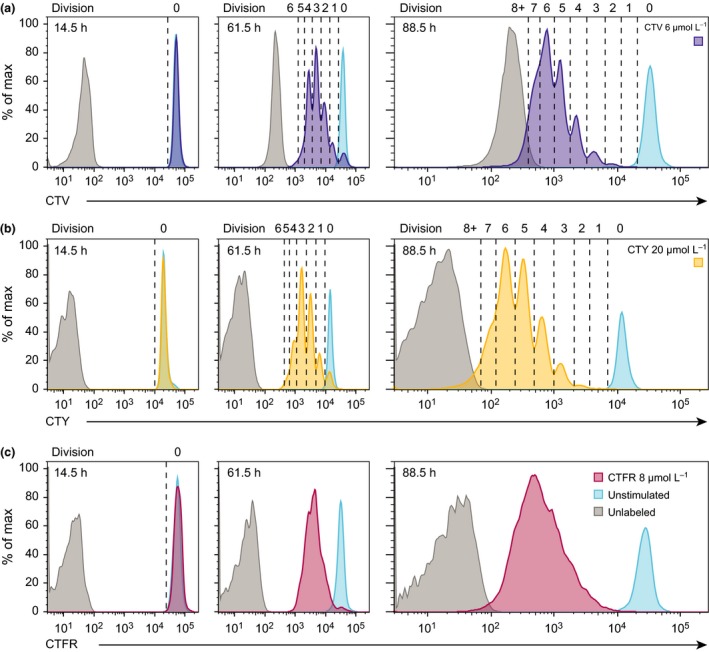
CTY shows greater sensitivity for division tracking than CTV and CTFR. Naïve B cells were purified, left unlabeled (gray) or labeled with CTV, CTY or CTFR, as indicated, and cultured in the presence (purple, yellow, or red) or absence (blue) of anti‐CD40 and IL‐4 for 3.5 days. Cells were harvested daily and the division profiles of stimulated cells for each cell tracking dye were analyzed for **(a)** CTV (purple), **(b)** CTY (yellow) and **(c)** CTFR (red). Where possible, division peaks were isolated, as indicated by dashed lines. Data are representative of 3 independent experiments where all 3 dyes were used in parallel. CTY, CellTrace Yellow; CTV, CellTrace Violet; CTFR, CellTrace Far Red.

Division peaks of activated cells labeled with CTV and CTY enabled clear peak resolution until autofluorescence was reached (Figure 2a and b), whereas CTFR peaks of cells under the same conditions were poorly defined, prohibiting analysis of individual division peaks using CTFR (Figure 2c). Owing to the increased distance between background autofluorescence and the division zero peak of labeled cells, we could clearly resolve more peaks with CTY than CTV; background autofluorescence began to interfere with peak segregation in division 8 for CTY, and by division 7 for CTV.

### Improved resolution of CTFR peaks is achieved by cell‐sorting a narrow peak of labeled cells

**Figure 3:**
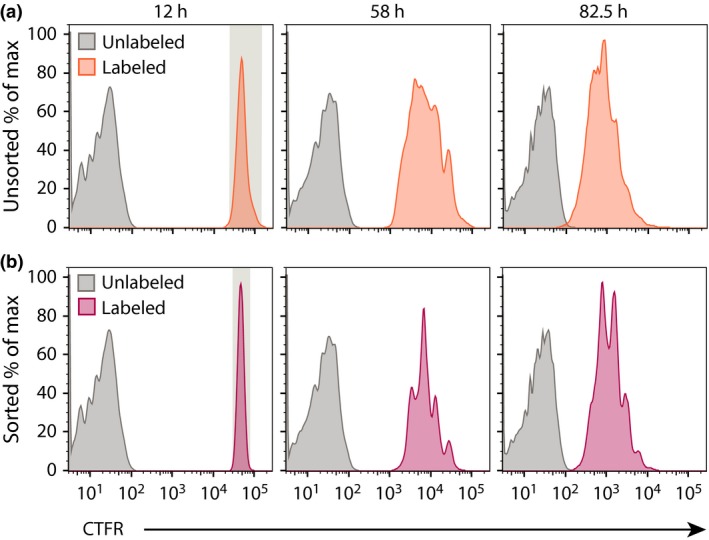
CTFR labeling is improved by pre‐sorting narrow peak of undivided cells. Murine splenic naïve B cells labeled with 7.5 μmol L^−1^ CTFR were cultured in anti‐CD40 and IL‐4 for 10 h, then **(a)** left unsorted (orange) or **(b)** sorted into a narrow undivided peak (red) before being plated into triplicate wells and analyzed at specified times after initial culture. Data are representative of 3 independent experiments. CTFR, CellTrace Far Red.

As noted above, CTFR was not suitable for division tracking as peaks were not well defined (Figure 2c), impeding the ability to confidently gate and segregate histograms into generations for analysis. Further scrutiny of the histogram profiles indicated that an initial broad, undivided peak was observed for CTFR (spanning up to 5‐fold range). Thus, despite even distribution of the dye among daughter cells, the peak for each subsequent generation remained equally broad, resulting in considerable overlap between consecutive generations. We sought to resolve this limitation using a method adopted by Nordon^33^, ^34^, ^35^ and others,^18^, ^36^, ^37^ sorting cells in the initial peak for a narrower range. This method successfully resulted in clear definition of subsequent generations (Figure 3), indicating dilution of this dye occurs with high fidelity and that the initial variation observed is principally due to the range of labeling of each initial cell.

### CTV and CTY do not affect cellular behaviors of human naïve and memory B cells

**Figure 4:**
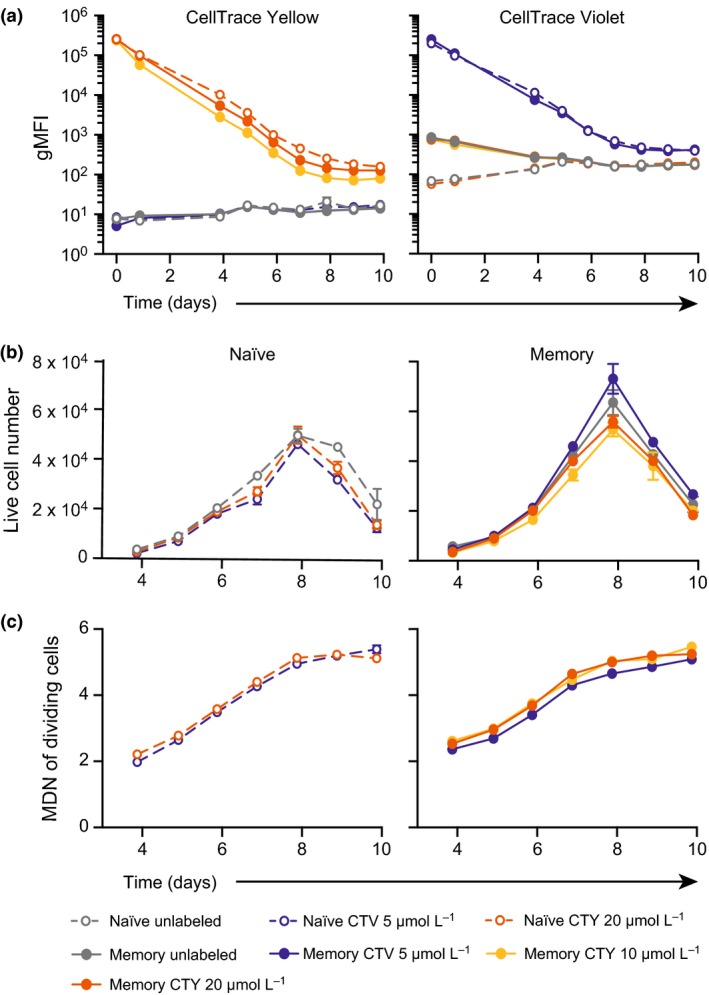
CTV and CTY‐labeling of human naïve and memory B cells do not impact cellular behaviors. Human splenic naïve and memory B cells were labeled with CTV or CTY as indicated and cultured in the presence of CD40L (100 ng mL^−1^) and IL‐21 (50 ng mL^−1^). After 4 days, cells were harvested, washed and re‐stimulated under the same conditions and **(a)** the gMFI of CTV and CTY assessed after 24 h of re‐culture, then daily from days 4–10. **(b)** The number of live cells present at each time point was assessed in cultures of human naïve (left) and memory (right) B cells. Mean live cell numbers ± SEM of replicate wells are shown. No significant differences were observed between the live cell number of unlabeled cells or cells labeled with each division tracking dye (Kruskal–Wallis test with Dunn's multiple comparisons, GraphPad Prism). **(c)** The mean division number of dividing cells was assessed in cultures of activated naïve (left) and memory (right) B cells. No significant differences were observed between the mean division number of unlabeled cells or cells labeled with each division tracking dye (One‐Way ANOVA, GraphPad Prism). Data are representative of (A) two (CTV) and at least 5 (CTY) independent experiments using cells from different donors and (B) and (C) 3 independent experiments. CTY, CellTrace Yellow; CTV, CellTrace Violet; CTFR, CellTrace Far Red; gMFI, geometric mean fluorescence intensity.

Having determined that CTY exhibited a number of superior features over CTV and CTFR for tracking murine naïve B cells, we sought to extend this observation to human naïve B cells, as well as human memory B cells, which are known to be more difficult to analyze using division tracking dyes.^38^ This difficulty has been attributed to their non‐uniform shape and size.^39^ As observed for murine B cells, the gMFI of unlabeled human B cells was considerably lower in the CTY channel (4–5 decades between unlabeled and undivided labeled cells) than in the CTV channel (3 decades between unlabeled and undivided labeled cells; Figure 4a), when cytometer voltages were optimized to labeled cells. The gMFI of unlabeled naïve and memory B cells in the CTY channel were similar, and remained stable over the course of the culture. In the CTV channel, the gMFI of unlabeled naïve cells increased upon activation (day 4) and subsequently remained stable over the course of the culture. The gMFI of unlabeled memory cells decreased over time in culture. The log value of gMFI of labeled cells diminished in a stable manner over time as the populations progressed through divisions, consistent with halving of fluorescence as the cells divided (Figure 4a).

The kinetics of human naïve and memory B cell responses to stimulation with CD40L and IL‐21 were comparable between cells labeled with CTV (5 μmol L^−1^), CTY (at both 10 μmol L^−1^ and 20 μmol L^−1^), and those that were not labeled. Proliferation of human naïve and memory B cells, measured by live cell number over time, was not significantly different between CTV, CTY, and unlabeled cells (Kruskal–Wallis test with Dunn's multiple comparisons, *P* > 0.05; Figure 4b). Naïve and memory B cell survival was also unaffected by CTV or CTY labeling (data not shown). The average population division level was quantified using mean division number of dividing cells (Figure 4c), and was not significantly different between naïve or memory cells labeled with CTV, CTY or no dye (One‐way ANOVA, *P* > 0.05). Together, these data demonstrate that the kinetics of human naïve and memory B cell responses were unchanged whether the cells were labeled with CTV, CTY, or left unlabeled.

### CTY allows the accurate resolution of an additional round of cell division

**Figure 5:**
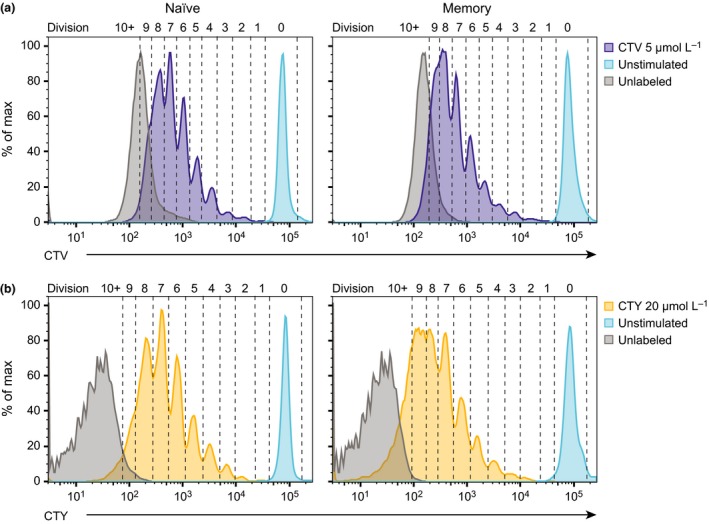
CTY increases the number of cell divisions that can be defined. Human naïve and memory B cells were labeled with CTV (5 μmol L^−1^) or CTY (20 μmol L^−1^) and cultured in the presence or absence of CD40L and IL‐21 for 7 days, and re‐stimulated after 4 days. Division profiles with the number of cell divisions is shown for **(a)** CTV‐labeled (purple) and **(b)** CTY‐labeled (yellow) naïve (left) and memory (right) B cells after 7 days of culture. For each plot, unlabeled, activated cells (gray) represent autofluorescence for each cell tracking dye, and labeled, unstimulated cells (blue) indicate the fluorescence level of undivided cells. Data are representative of 2 independent experiments. CTY, CellTrace Yellow; CTV, CellTrace Violet.

We observed that the peak of each division was more defined for both human naïve and memory B cells when labeled with CTY, compared to cells labeled with CTV, with little overlap observed between division peaks. This was particularly apparent for memory B cells, where we were able to resolve up to 9 cell divisions when labeled with CTY, not previously possible with CTV‐labeling (Figure 5). Memory B cells and other cell types with high intrinsic morphological variation typically produce ill‐defined peaks using CTV and other cell tracking dyes,^36^, ^38^ presumably due to the non‐uniform shape and size of these cells compared to naïve B cells (and T cells) which are relatively small and uniform. This may also be true for other cell types that have poor resolution using CFSE or CTV staining.

Cells labeled with CTV reached fluorescence levels indistinguishable from background fluorescence of activated cells by division 7–8, compared to division 9 using CTY (Figure 5). This observation has significant implications for the capacity to use these dyes in studying events that occur after several rounds of cell division, such as differentiation into antibody‐secreting cells, and isotype switching of naïve B cells.

We quantified the proportion of overlap between division peaks of cells labeled with CTV or CTY, and the autofluorescence levels of unlabeled cells in each channel by counting the number of pixels from the relevant regions of each histogram (Supplementary figure 2). For CTV‐labeled cells, the levels of autofluorescence began to overlay with division peaks at division 6, and overlap was 100 percent by the 10th round of cell division. In contrast, the intersection between autofluorescence and division peaks for the same time point, when cells were labeled with CTY, occurred 3–4 divisions later, at division 9 (for naïve) and division 10 (for memory). Although autofluorescence and division peaks completely overlapped after 10 divisions in naïve B cells, there was potential for further division slicing in memory B cells.

## Discussion

Cell tracking dyes including CFSE, CTV, and more recently, CTY and CTFR enable enumeration of the number of divisions a cell or population of cells has undergone. Analysis of cell division using these dyes has led to significant advances in our understanding of lymphocyte biology and immune response dynamics. In this report, we have outlined a number of significant advantages of utilizing new generation cell tracking dyes, and in particular, CellTrace Yellow (CTY).

We have demonstrated that CTV, CTY and CTFR are each compatible with labeling of murine naïve and human naïve and memory B lymphocytes, and at working concentrations impart no detrimental impact on the cellular behaviors of these cells, including proliferation, survival, or division rates. We have shown that the background fluorescence levels of unlabeled cells in the CTY and CTFR channels is significantly lower than that of the CTV channel, which allows a greater number of divisions to be confidently enumerated when cells are labeled with CTY and CTFR. Further, CTY peaks are distinct and more easily resolved than those of CTV and CTFR. In addition to clearer peak resolution, especially at the low end of the spectrum (i.e., improved resolution of later divisions), CTY also enables enumeration and analysis of an additional round of cell division, compared to CTV, owing to a greater dynamic range between labeled cells and autofluorescence in the channel used to detect CTY, and a higher threshold of toxicity, which together permit a higher‐intensity of initial staining.

These properties of CTY represent a significant advantage over CFSE and CTV, previously the most commonly used cell tracking dyes. While CTV is sufficient for tracking up to 7 divisions of cells such as naïve T and B cells, which are small, round cells of homogenous morphology, it may be less appropriate for investigation of populations that are non‐homogenous in size and/or shape, including memory B cells, plasma cells, tumor cells or other morphologically irregular cells. Isolating division information for as many divisions as possible is critically important when investigating events that primarily occur after multiple rounds of division, in particular division‐linked effector cell differentiation. Moreover, memory B cells are primed to proliferate faster and attain more divisions earlier than naïve B cells.^40^ As such, the ability to track a larger number of divisions, as afforded by CTY labeling, facilitates a more accurate analysis of memory B cell behaviors and kinetics, and is likely to allow late division‐linked changes to be better investigated.

With any cell division tracking dye, peaks compress as they reach autofluorescence according to a predictive formula.^9^, ^41^, ^42^ This compression makes it more challenging to distinguish individual peaks as generation numbers increase, and peak‐fitting using automated software becomes less accurate as cells divide. As a result, a division tracking dye utilizing a channel with a lower level of autofluorescence, such as CTY, allows more accurate estimates of the number of cells after a greater number of divisions. Thus, CTY can facilitate existing automated proliferation analysis tools to estimate cell numbers in each generation, and extract proliferation, survival, and differentiation information as required for many quantitative applications.^10^, ^12^, ^15^, ^16^, ^17^

During our investigation of recently available cell division tracking dyes, we determined that poor CTFR peak resolution was improved by sorting labeled cells prior to first division for a narrow fluorescence range, generating clear division peaks in subsequent analyses. Thus, poor peak resolution can be attributed to broad, inconsistent labeling of the initial cell population, presumably due to slight dye uptake differences between CTV and CTY, created by their unique chemistry. Given the strong heritability once labeled, in theory, the variance of labeled starting populations could be as narrow as flow cytometric sorting will allow, which may permit resolution of additional division peaks before the generations overlap as they reach autofluorescence; particularly for CTFR and CTY dyes. For instance, it may be possible to visualize significantly more than 10 rounds of cell division and potentially capture a more complete immune response. Cell number would be a limiting factor in this experimental design, hence in practice, sorting to narrow labeling variation must be balanced with the availability of the target cell type, and the cell numbers required for the experiment of interest. This technique may be suitable for experiments such as the *in vivo* transfer of sorted cell populations, where only low cell numbers are required, but high fidelity is particularly beneficial.

Reducing initial labeling variation has potential applications outside of flow cytometry, such as for identifying division numbers by microscopy, especially when cells commence dividing prior to imaging, or when intervals between time‐lapse images are too great for accurate lineage‐tracking. Live cell imaging typically requires low light levels to prevent phototoxicity,^43^ which consequently constrains the dynamic range for detecting fluorophore intensity as compared to flow cytometry. Allocating imaged cells to the correct division number may be performed with greater confidence were each division to occupy only a narrow range of fluorescence values. Due to the low energy excitation wavelengths used for CTY or CTFR, repeat exposures using these dyes as opposed to the high‐energy wavelengths required for CTV significantly reduces the risk of photodamage, thus making CTY and CTFR well suited for live‐cell imaging.^43^

The availability of a range of cell tracking dyes with unique fluorescence properties now also gives users the ability to perform multiplex staining – that is, label with different combinations of division tracking dyes to create uniquely labeled cells that can subsequently be followed *in vitro* or *in vivo*.^17^ This has been used for CD8 +T cell cytotoxicity and regulatory T cell suppression assays *in vitro* and *in vivo,* 6, 22, 23, 44 as well as for tracking the division outcomes of single lymphocyte families.^17^, ^41^, ^45^ The availability of additional dyes on other emission spectra allow further combinations to be developed for such applications. Multiple cell division tracking dyes could also be used to investigate beyond ten divisions, by initially sorting for cells within a single division peak, before re‐labeling with a different cell tracking dye at a later time. This could be of particular benefit in the study of highly proliferative cell populations such as germinal centre B cells or hematopoietic progenitor cells.

We have demonstrated here that CTY has significant advantages over other division tracking dyes, allowing more divisions to be tracked, the option to multiplex‐label with ever increasing combinations of dyes, and many other possible applications. While it must be considered that the broad emission spectra of CTY means it will be detected by many of the channels excited by the yellow‐green laser, a caveat that must be taken into consideration when designing any dye‐labeling panels, users now also have the ability to choose a cell division tracking dye that complements other requirements of their experimental design. A wide range of fluorophores are becoming ever more available, with more in development, for flexible, multi‐color panels using monoclonal antibodies, fluorescent reporter animal models, and cell lines. The use of CTY should be facilitated where possible to take advantage of its enhanced division tracking features.

## Methods

### Mice

C57BL/6 mice were bred and maintained under specific pathogen‐free conditions in the Walter and Eliza Hall Institute (WEHI) animal facility (Parkville, Victoria, Australia). For initial proliferation dye titrations, naïve B cells were isolated from pooled splenocytes of two female mice, euthanized between 7 and 9 weeks of age. For subsequent experiments, both male and female mice were used, and euthanized between 7 and 16 weeks of age for organ collection. All animal experiments were conducted in compliance with the Australian code for the care and use of animals for scientific purposes and were approved by the WEHI Animal Ethics Committee.

### Murine B cell culture

Murine, naïve, splenic B cells were purified by first using a percoll (GE Healthcare, IL, USA) gradient (80/65/50% in PBS, cells collected from 80/65% interface), followed by negative isolation with a B cell isolation kit (Miltenyi Biotech, Bergisch Gladbach, Germany). Once labeled (see “Cell labeling with division tracking dyes” below), cells were cultured in Advanced B cell medium (Advanced RPMI‐1640, supplemented with 5% (vol/vol) FBS, 10 mmol L^−1^ HEPES, 100 U mL^−1^ penicillin, 100 μg mL^−1^ streptomycin, 2 mmol L^−1^ GlutaMAX (Gibco, Thermo Fisher Scientific, MA, USA) and 50 μmol L^−1^ β‐2‐mercaptoethanol (Sigma‐Aldrich, MO, USA)) at 10^4^ cells per 200 μL, and stimulated with 10 μg mL^−1^ anti‐CD40 antibody (clone 1C10; WEHI Antibody Facility) and 500 U mL^−1^ murine recombinant IL‐4 (WEHI). Triplicated cultures were plated into 96‐well flat‐bottomed plates (BD Falcon, NJ, USA) and incubated at 37°C with 5% CO_2_.

### Human B cell culture

Healthy human spleens from cadaveric organ donors were obtained from Victorian Cancer Biobank. All studies were approved by WEHI Human Research Ethics Committee (Project 10/02). Naïve and memory B cells were isolated from splenocytes with anti‐human CD20‐FITC (BD Biosciences, CA, USA., catalog number 555622) and CD27‐BV421 (BD Biosciences, catalog number 562513) and cell sorting for CD20^+^CD27^−^ (naïve) and CD20^+^CD27^+^ (memory) B cells. Cells were sorted on a FACS Aria Fusion flow cytometer (BD Biosciences). The post‐sort purity for each population was >98%. Once labeled with division tracking dyes, purified naïve and memory B cells were cultured in B cell medium (RPMI‐1640, supplemented with 10% (vol/vol) FBS, 10 mmol L^−1^ HEPES, 100 U mL^−1^ penicillin, 100 μg mL^−1^ streptomycin, 2 mmol L^−1^ GlutaMAX, 0.1 mmol L^−1^ non‐essential amino acids, 1 mmol L^−1^ sodium pyruvate (Gibco, Thermo Fisher Scientific), 0.1 mg mL^−1^ Normocin (InVivogen, CA, USA.) and 50 μmol L^−1^ β‐2‐mercaptoethanol (Sigma‐Aldrich)), and were stimulated with megaCD40L (100 ng mL^−1^; Sapphire Biosciences, NSW, Australia) and recombinant human IL‐21 (50 ng mL^−1^; Peprotech, NJ, USA). Cells were plated in 96‐well round‐bottom plates (BD Falcon) at 5 x 10^4^ cells per well for 4 days at 37°C with 5% CO_2_. After 4 days, cell cultures were diluted 1 in 20, re‐stimulated in fresh B cell medium, and cultured under the same conditions for an additional 6 days. Cells were harvested daily as described below**.**

### Cell labeling with division tracking dyes

Purified murine or human B cell populations were suspended at a density of 2 × 10^7^ cells mL^−1^ in PBS/0.1% bovine serum albumin (BSA) in preparation for cell labeling. CellTrace Violet (Molecular Probes, Thermo Fisher Scientific, catalog number C34557), CellTrace Yellow (catalog number C34567), and CellTrace Far Red (catalog number C34564) were prepared at twice the final concentration in PBS/0.1% BSA, and a volume equivalent to each cell suspension was added to each tube immediately prior to incubation in a 37°C water bath for 20 min. The staining was stopped with a 1 in 10 dilution in cold B cell media, washed twice, and cells were counted before being placed into culture.

### Flow cytometry

Replicate wells were analyzed as indicated over 5 days (for murine B cell cultures) or 10 days (for human B cell cultures) using a BD LSRFortessa X20 (Becton Dickinson, NJ, USA.). CTV was excited with the 405 nm laser and detected with the 450/50 bandpass filter. CTY was excited with a 561 nm laser and detected with the 586/15 bandpass filter. CTFR was excited with a 635 nm laser and detected with a 670/30 bandpass filter (Table 1). A known number of beads (CaliBRITE rainbow calibration particles, BD Biosciences) were added to samples immediately prior to analysis for calculating cell numbers, and propidium iodide (0.2 μg mL^−1^, Sigma) or hydroxystilbamidine (methanesulfonate, 1 μg mL^−1^, Invitrogen, Thermo Fisher Scientific) was included for dead cell exclusion in murine and human experiments, respectively.

### Cell division analysis

The absolute number of cells was determined by calculating the ratio of CaliBRITE Rainbow beads to live cells in each sample. Cell division analysis was performed using FlowJo software (Tree Star, OR, USA). Analysis of division peaks was performed using log‐fluorescence and the proportion of cells in each division calculated from division profiles and bead counts as previously described.^16^ Cohort number,^46^ as a measure of the number of founding cells, and their descendants, still present in culture, was calculated by dividing the live cell number by two to the power of division number, and mean division number was calculated for divided cells (i.e., excluding those cells in undiluted division peak) by multiplying the cohort number per division by the division number, and dividing the sum of these numbers by the total cohort number, as previously described.^16^, ^46^ Data were graphed and statistical analyses performed using Prism software (GraphPad, CA, USA). For comparison of human B cell kinetics between division tracking dyes, One‐way ANOVA was used (GraphPad Prism).

### Staining index

Staining Index values were calculated according to the equation by Maecker *et. al,* 32 (mean_labeled_–mean_background_)/(2 × SD_background_), where the geometric mean fluorescence intensity was used here as the mean, and robust standard deviation used for the SD, as determined by FlowJo (Tree Star).

### Quantifying autofluorescence

Calculation of the intersection between division peaks and autofluorescence of each cell tracking dye was performed using Adobe Photoshop. The number of pixels in the overlapping area between the autofluorescence peak of unlabeled cells and each division peak of labeled cells were enumerated for each cell tracking dye to calculate a percentage.

## Conflict of Interest

The authors declare no conflict of interest.
