# Supplementary material for: Superior properties of CellTrace Yellow™ as a division tracking dye for human and murine lymphocytes
Source: Immunol Cell Biol. 2017 Dec 15;96(2):149–59. doi: 10.1111/imcb.1020 (PMC6446909; doi:10.1111/imcb.1020)
Supplement: Supplementary file 3 [file IMCB-96-149-s003.docx]

*Supplementary Table 1: Staining Index (SI) of division tracking dyes at the indicated times after culture*

| **Division tracking dye** | **14.5 h** | **61.5 h** | **88.5 h** |
| --- | --- | --- | --- |
| CellTrace Violet 6 μM | 1110.6 | 262.7 | 206.7 |
| CellTrace Yellow 20 μM | 592.5 | 347.0 | 296.7 |
| CellTrace Far Red 8 μM | 1126.7 | 564.0 | 454.0 |
